# A Hypoxic Environment Attenuates Exercise-Induced Procoagulant Changes Due to Decreased Platelet Activation

**DOI:** 10.1055/s-0039-1692991

**Published:** 2019-07-22

**Authors:** Cécile H. Kicken, Lisa N. van der Vorm, Suzanne Zwaveling, Evi Schoenmaker, Jasper A. Remijn, Dana Huskens, Bas de Laat

**Affiliations:** 1 Department of Anesthesiology, Maastricht University Medical Center, Maastricht, The Netherlands; 2 Synapse Research Institute, Maastricht, The Netherlands; 3 Department of Clinical Chemistry and Hematology, Gelre Hospitals, Apeldoorn, The Netherlands; 4 Department of Biochemistry, Cardiovascular Research Institute Maastricht, Maastricht University, Maastricht, The Netherlands; 5 Department of Clinical Chemistry, Meander Medical Center, Amersfoort, The Netherlands

**Keywords:** exercise, hypoxia, thrombosis, thrombin generation, platelet activation

## Abstract

**Introduction** Although physical exercise is protective against cardiovascular disease, it can also provoke sudden cardiac death (exercise paradox). Epidemiological studies suggest that systemic hypoxia at high altitude is a risk factor for venous thromboembolism. Forthcoming, this study investigated the effect of repeated exercise at high altitude on blood coagulation, platelet function, and fibrinolysis.

**Methods** Six trained male volunteers were recruited. Participants ascended from sea level to 3,375 m altitude. They performed four exercise tests at 65 to 80% of their heart-rate reserve during 2 hours: one time at sea level and three times on consecutive days at 3,375 m altitude. Thrombin generation (TG) was measured in whole blood (WB) and platelet-rich and platelet-poor plasma. Coagulation factor levels were measured. Platelet activation was measured as αIIbβ3 activation and P-selectin expression. Fibrinolysis was studied using a clot-lysis assay.

**Results** Normoxic exercise increased plasma peak TG through increased factor VIII (FVIII), and increased von Willebrand factor (VWF) and active VWF levels. Platelet granule release potential was slightly decreased. After repetitive hypoxic exercise, the increase in (active) VWF tapered, and there was no more distinct exercise-related increase in peak. Platelet aggregation potential and platelet-dependent TG decreased at high altitude. There were no effects on fibrinolysis upon exercise and/or hypoxia.

**Conclusion** Strenuous exercise induces a procoagulant state that is mediated by the endothelium, by increasing VWF and secondarily raising FVIII levels. After repetitive exercise, the amplitude of the endothelial response to exercise diminishes. A hypoxic environment appears to further attenuate the procoagulant changes by decreasing platelet activation and platelet-dependent TG.

## Introduction

Mountaineering involves repetitive physical exercise in a hypoxic environment. Although physical exercise is generally protective against cardiovascular events, there are numerous reports of exercise-related thromboembolic and cardiovascular events. 1 2 3 Additionally, it has been found that high altitude increases the risk of venous thromboembolism (VTE). 4 5 6 7 Moreover, cardiac arrest at high altitude due to coronary thrombosis has been reported. 8

The risk of cardiovascular and thromboembolic events is partially determined by hypercoagulability. Exercise is known to exert many effects on the hemostatic system, mainly through endothelial activation, which causes von Willebrand factor (VWF) and factor VIII (FVIII) elevation, platelet hyperreactivity, increased thrombin generation (TG) as well as elevated fibrinolytic markers. 9 10 11 All in all, these changes result in a shift toward a transient prothrombotic state. 12 The influence of hypoxia on hemostasis is less well characterized. A few studies found that systemic hypoxia influences hemostasis through the elevation of FVIII levels, as that occurs in response to strenuous exercise. 13 14 It has long been known that elevated FVIII levels are a risk factor for VTE, likely by increasing TG. Mechanistically, hypoxia may induce this FVIII-dependent increase in TG via alteration of the redox status of the blood, i.e., by inducing reactive oxygen species formation. 15 Supporting this, the anticoagulant vitamin E prevented increases in both FVIII and TG following 2 hours of exposure to normobaric hypoxia. 14

Because both hypoxia and exercise induce hypercoagulability, it seems likely that exercise amplifies the altitude-induced hypercoagulability. However, several studies found that hypoxia actually attenuates the exercise-induced hypercoagulable response, mainly through depression of platelet activation. 16 17 18 19 It has never been investigated whether this effect persists after repeated exercise at high altitude. Forthcoming, this pilot study aimed to investigate the effect of repeated cycling at 3,375 m altitude on TG, platelet activation, and fibrinolysis.

## Methods

### Inclusion of Subjects

This study was approved by the medical research ethics committee from Maastricht University (METC azM/UM, reference NL61217.068.17), was monitored by the Clinical Trial Center Maastricht, and met all standards of the Declaration of Helsinki (version 10, 2013). The primary endpoint was WB TG peak height at high altitude. Group size was calculated based on data from a previous high-altitude study, 20 in which the peak height was 139 nM at sea level and rose to 241 nM at 2,045 m altitude; the estimated standard deviation (SD) was 40 nM. With eight pairwise comparisons and α = 0.05, at least four subjects needed to be recruited to achieve a power of 80%. 21 To allow dropouts without underpowering the study, a total of six trained and healthy men were included in this study. Exclusion criteria were cardiovascular disease, pulmonary disease, impaired mobility, known coagulation disorders, and medication interfering with coagulation (heparins, vitamin K antagonists, new oral anticoagulants, or NSAIDs). After informed consent, but before inclusion, all participants passed a medical assessment by an independent cardiologist, consisting of history taking, vital signs (peripheral oxygen saturation [SpO _2_ ], heart rate [HR], blood pressure), auscultation of heart and lungs, and an exhaustive ergometry test according to the Bruce protocol. 22 Prior to the study, all participants were exercising (four out of six cycling, subject 3 fitness/running, and subject 5 hockey) on a regular basis, for an average of 5.6 hours per week (SD: 1.3 hours).

### Exercise and Altitude Protocol

**Fig. 1:**
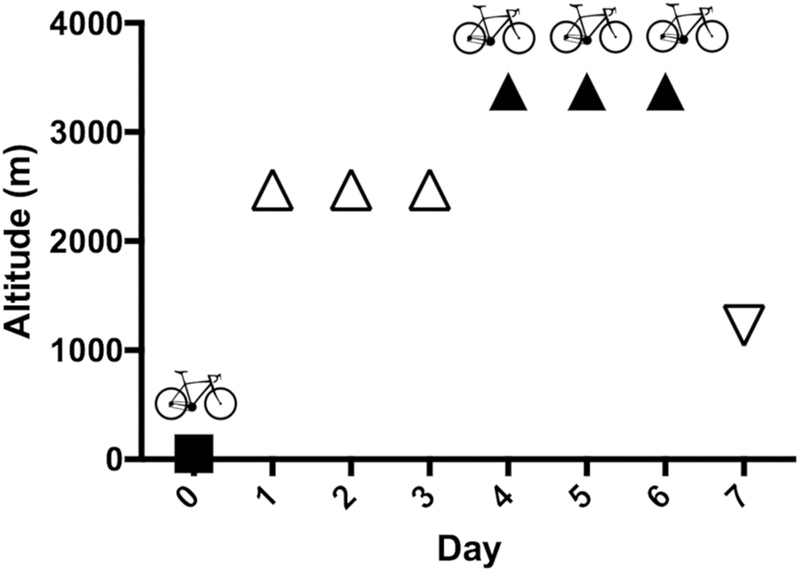
Schematic representation of the study set-up. Participants performed an exercise test on four occasions: one time at 50 m altitude (normoxic exercise), and three times on 3 consecutive days at 3,375 m altitude (hypoxic exercise). Before the hypoxic exercise tests, participants acclimatized to the altitude for 3 days at 2,475 m altitude. After the last hypoxic exercise test, participants descended to 1,225 m altitude.

The altitude and exercise protocol is shown in Fig. 1 . Vigorous exercise can be defined as 60 to 85% of HR reserve (HRR), where HRR = HR _max_ − HR _rest_ . 23 These boundaries were calculated using data from the exhaustive ergometry test, using the following equation: exercise HR = HR _rest_ + 0.6/0.85 × HRR. The participants delivered monitored physical exercise by cycling for 2 hours on a racing bike mounted on a stationary frame (Tacx Blue Twist, Wassenaar, the Netherlands). HR was monitored every 10 minutes using a chest strap (Polar FT1 sports watch, Kempele, Finland), and participants were encouraged to keep their HR between their predefined HRR boundaries to ensure adequate exercise intensity. The exercise test was performed on four different occasions: one time at 50 m altitude (normoxic exercise), and three times on 3 consecutive days at 3,375 m altitude (hypoxic exercise). Between the baseline measurement and the hypoxic exercise tests, participants acclimatized to the altitude. First, they stayed for 3 days at 2,473 m altitude, where daily activities but not exercise were allowed. On the third day, they ascended further to 3,375 m altitude. After one night at 3,375 m altitude (day 4), the first hypoxic exercise tests were performed, followed by the second and third hypoxic exercise tests on the next 2 days (day 5 and 6). The test room and laboratory were set up in a ventilated mountain cabin at room temperature. Participants stayed overnight at 3,375 m altitude unless there was a reason for earlier descent. On day 6, after completion of the last exercise test at 3,375 m altitude, the participants descended immediately to 1,224 m altitude.

### Lake Louise Acute Mountain Sickness Questionnaire

To record signs of acute mountain sickness (AMS), participants filled out a questionnaire based on the Lake Louise Consensus on the Definition of Altitude Illness 24 at 50 m altitude, and every morning at 2,473 m and 3,375 m altitude.

### Blood Collection

Blood was drawn before and after every exercise test by venipuncture of the antecubital vein. The blood was aseptically drawn in Vacutainer tubes (Greiner Bio-One) containing 3.2% sodium citrate (nine volumes blood, one volume anticoagulant). To inhibit contact activation, a separate 3 mL citrate tube with added corn trypsin inhibitor (CTI, Enzyme Research Laboratories, South Bend, Indiana, United States; final concentration 50 μg/mL) was drawn. Any particularities (bleeding, bruising, difficult puncture, and hemolysis) were noted. The blood was kept at room temperature (21°C) until use. Platelet-rich plasma (PRP) was obtained by centrifuging the blood at 240 *g* for 15 minutes. Platelet-poor plasma (PPP) was obtained by centrifuging the blood twice at 2,830 *g* for 10 minutes and immediately placed on dry ice (−80°C) for later analysis.

### Biochemical Markers

Albumin as a marker for dehydration, creatinine and urea as markers for kidney function, lactate as a marker for anaerobic metabolism, and C-reactive protein as a marker for inflammation were measured with an ARCHITECT ci8200 (Abbott Diagnostics, Lake Forest, United States).

### Blood Count

Red blood cell count (RBC, ×10 ^12^ /L), hematocrit (Ht, L/L), hemoglobin levels (Hb, mmol/L), mean corpuscular Hb concentration (MCHC, mmol/L), white blood cell count (WBC, ×10 ^9^ /L), granulocyte count (GR, ×10 ^9^ /L), lymphocyte count (LY, ×10 ^9^ /L), monocyte count (MO, ×10 ^9^ /L), and platelet count (PC, ×10 ^9^ /L) were determined with a Coulter LH-750 analyzer (Beckman Coulter, Brea, United States) within 2 hours after venipuncture in the citrated WB. Values were corrected for 10% dilution by the citrate solution present in blood tubes.

### Coagulation Factor Analysis

Antithrombin (AT), FVIII concentration (FVIII:C), VWF antigen (VWF:Ag), and fibrinogen levels were measured by batch analysis in PPP by the STA-R Evolution (Diagnostica Stago, Leiden, the Netherlands). Active VWF was measured in plasma by an enzyme-linked immunosorbent assay (ELISA), as described previously. 25 Normal pooled plasma (NPP) was used as a standard in every plate and sample results were normalized (%) to NPP on the same plate.

### Whole Blood Thrombin Generation

The WB TG assay was performed within 2 hours after blood collection, triggered with 0.5 pM tissue factor (TF) or without added TF. The method has been described previously. 26 For the high-altitude measurements, the necessary equipment was installed at 3,375 m altitude in a ventilated room, to allow measurement in fresh undisturbed blood. In short, 30 µL citrated WB was mixed with 10 µL rhodamine substrate (thrombin specific fluorogenic substrate, 1.8 mM) and activated with 20 µL of either a mix of CaCl _2_ (50 mM) and HEPES buffer containing 5 mg/mL BSA (BSA5) with/without TF, or a calibrator (α _2_ -macroglobulin–thrombin complex, in-house prepared, 300 nM thrombin activity). The sample was mixed, and 5 µL was transferred immediately onto a paper disk (Whatman GmbH, Dassel, Germany) in Immulon 2HB flat-bottom 96-well plates (Thermo Scientific, Waltham, United States), and covered with 40 µL mineral oil (USB Corporation). TG was measured in triplicate in a 96-well plate fluorometer (Ascent reader, Thermo Labsystems Oy, Helsinki, Finland) equipped with a 485/538 nm filter set (excitation/emission). Samples were measured for 40 minutes at 37°C. Raw data were converted into thrombograms as described previously. 27 Parameters derived from the TG curve were peak height (peak, nM) and endogenous thrombin potential (ETP, nM·min).

### Calibrated Automated Thrombinography in Plasma

The calibrated automated thrombinography assay was performed on the spot in PRP and later by batch analysis in snap-frozen PPP. The method has been described previously. 28 For the measurements of PRP TG at high altitude, the necessary equipment was installed at 3,375 m altitude in a ventilated room. TG was determined in triplicate at 37°C in PRP after addition of 1 pM TF (PRP-reagent, Thrombinoscope, Maastricht, the Netherlands). TG was also determined in triplicate at 37°C in PPP after addition of 1 pM TF plus 4 μmol/L phospholipids (PPP-low reagent), in PPP after addition of 5 pM TF plus 4 μmol/L phospholipids (PPP-reagent), and in citrate-CTI anticoagulated PPP (PPP-low reagent), according to the manufacturer's instructions. A sample of NPP was added on each 96-well plate for normalization, to obtain acceptable interassay variations. 29 As a calibrator, α _2_ -macroglobulin–thrombin complex (α _2_ M-T, ± 600 nM thrombin activity, Thrombinoscope, Maastricht, the Netherlands) was used. Z-Gly-Gly-Arg-AMC (FluCa kit, Thrombinoscope, Maastricht, the Netherlands) was used as a fluorogenic substrate. The thrombograms were measured in a fluorometer equipped with a 390/460 nm filter set (excitation/emission) and a dispenser. Immulon 2HB round-bottom 96-well plates (Thermo Scientific, Waltham, United States) were used. A dedicated software program (Thrombinoscope, Maastricht, the Netherlands) calculated the thrombograms. Parameters derived from the TG curve were the peak (% of NPP) and ETP (% of NPP).

### Platelet Activation test in Whole Blood

The flow-cytometric platelet activation test in WB was performed as described previously. 30 Thrombin receptor activator peptide (TRAP-6, final concentration 30 µM, SFLLRN, H-2936; Bachem, Germany) and collagen-related peptide (CRP, final concentration 5 µg/mL, a kind gift of Prof. Farndale, University of Cambridge, United Kingdom) were used as platelet agonists. Moreover, an unstimulated control condition without agonist was included. The reaction mixtures contained three antibodies: APC-conjugated CD42b, (BD Bioscience), PE-conjugated anti-P-selectin, and fluorescein isothiocyanate (FITC)-conjugated PAC-1 against activated αIIbβ3 (BD Pharmingen, Franklin Lakes, United States). WB was preheated at 37°C for 10 minutes and the tests were performed at 37°C. WB was diluted 1:4 in HBS and 5 µL of this diluted blood were added to each reaction mixture. After exactly 20 minutes of incubation at 37°C, reactions were stopped by adding 250 µL fixation solution (137 mmol/L NaCl, 2.7 mmol/L KCl, 1.12 mmol/L NaH _2_ PO _4_ , 1.15 mmol/L KH _2_ PO _4_ , 10.2 mmol/L Na _2_ HPO _4_ , 4 mmol/L EDTA, and 0.5% formaldehyde).

Flow cytometry was used to discriminate platelets from other cells, using the forward and sideward scatter pattern and by gating on the CD42b positive cells. Fluorescence intensity in the FITC gate and PE gate was selected to determine activated αIIbβ3 and P-selectin density, respectively, and results were expressed as median fluorescence intensity.

### Clot Lysis Assay

To examine fibrinolysis, a turbidimetric clot lysis assay was performed in PPP using tissue-plasminogen activator (t-PA, Actilyse, Boehringer Ingelheim, Germany). Samples were tested in duplicate and preheated at 37°C for 10 minutes. A total of 80 µL of PPP was spiked with 20 µL of a TF/BSA5 solution (final concentration: 1 pM) plus phospholipids (final concentration: 4 μmol/L). Fibrin clot formation was started by adding 20 μl of a preheated CaCl _2_ /BSA5 solution (final concentration: 16.7 mM) with t-PA (final concentration: 100 IU). Optical density was measured at 405 nm with 20-second intervals during 1 hour at 37°C using a plate reader (SpectraMax M2, Molecular Devices, United States). All samples from one subject were measured simultaneously to avoid interassay error. Clot lysis time (CLT, min) was defined as the time from half-maximal fibrin formation to half-maximal degradation.

### Data Analysis

**Fig. 2:**
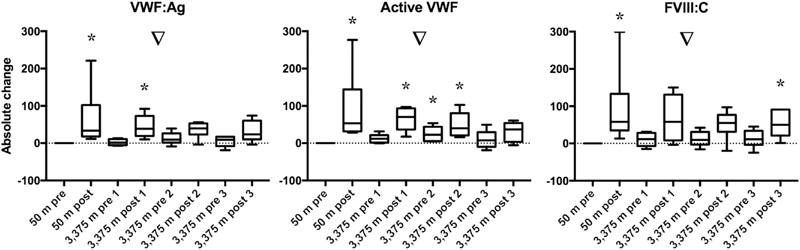
Normoxic and hypoxic exercises increased von Willebrand factor antigen (VWF:Ag) levels, active VWF, and factor VIII concentration (FVIII:C). Results are expressed as the absolute change of 50 m pre-exercise; a value above 0 indicates an increase, and a value below 0 indicates a decrease. ▿ = *p* < 0.05 (Friedman's test); * = *p* < 0.05 compared with zero (Wilcoxon signed-rank test).

Statistical analyses and figures were generated using Prism version 7 (GraphPad Software Inc., La Jolla, United States). Generally, data are represented as median ± interquartile range or [25th; 75th percentile]. Descriptive statistics were used to discover trends in the data. The data presented in Figs. 2 3 4 to 5 and Supplementary Tables S1 and S2 are expressed as absolute change compared with the first measurement at sea level; a value above 0 indicates an increase, and a value below 0 indicates a decrease. Absolute change, not percentage change, was chosen because this is more statistically powerful. 31 For statistical analysis, a nonparametric distribution was assumed because of the small sample size. The Wilcoxon signed-rank test was used to determine if the data presented in Figs. 2 3 4 to 5 and Supplementary Tables S1 and S2 were different from zero. Friedman's test with Dunn's post-hoc analysis was used to determine overall statistical significance of changes within participants. A *p* -value < 0.05 was considered significant.

**Fig. 3:**
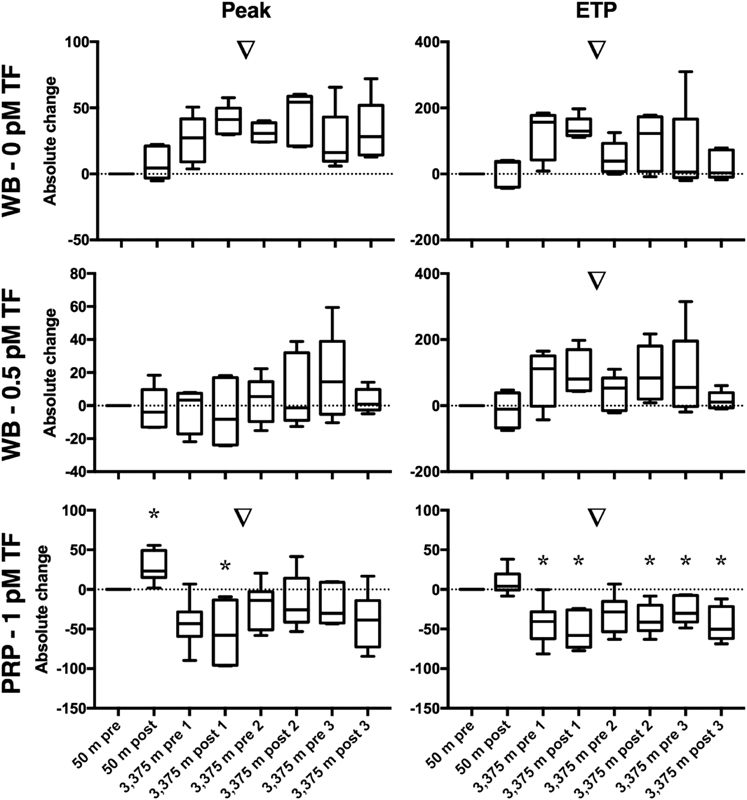
Influence of normoxic and hypoxic exercises on thrombin generation (TG) in whole blood (WB) and platelet-rich plasma (PRP). TG was measured before and after 2 hours of strenuous exercise, once at 50 m altitude and three times at 3,375 m altitude, in whole blood (WB) and PRP. Parameters derived from the TG curve are peak height (peak in nM or % of NPP) and endogenous thrombin potential (ETP in nM·min or % of NPP). *N* = 6, one measurement is missing (subject 5, WB TG post 3). Results are expressed as the absolute change of 50 m pre-exercise; a value above 0 indicates an increase, and a value below 0 indicates a decrease. ▿ = *p* < 0.05 (Friedman's test); * = *p* < 0.05 compared with zero (Wilcoxon signed-rank test).

**Fig. 4:**
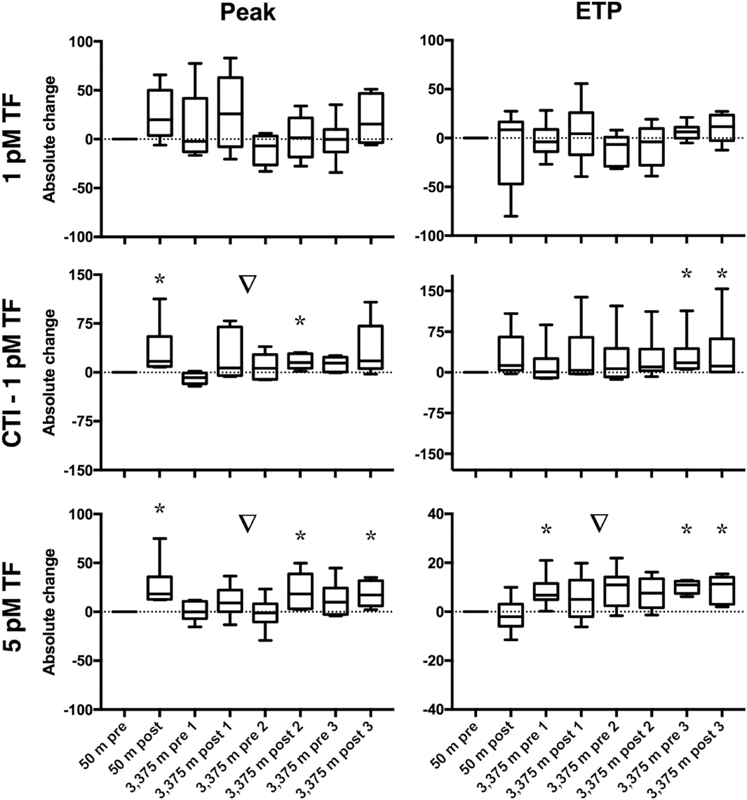
Influence of normoxic and hypoxic exercises on thrombin generation (TG) in platelet-poor plasma (PPP). TG was measured before and after 2 hours of strenuous exercise, once at 50 m altitude and three times at 3,375 m altitude, in PPP with and without added corn trypsin inhibitor (CTI). Parameters derived from the TG curve are peak height (peak % of NPP) and endogenous thrombin potential (ETP in % of NPP). *N* = 6, results are expressed as the absolute change of 50 m pre-exercise; a value above 0 indicates an increase, and a value below 0 indicates a decrease. ▿ = *p* < 0.05 (Friedman's test); * = *p* < 0.05 compared with zero (Wilcoxon signed-rank test).

**Fig. 5:**
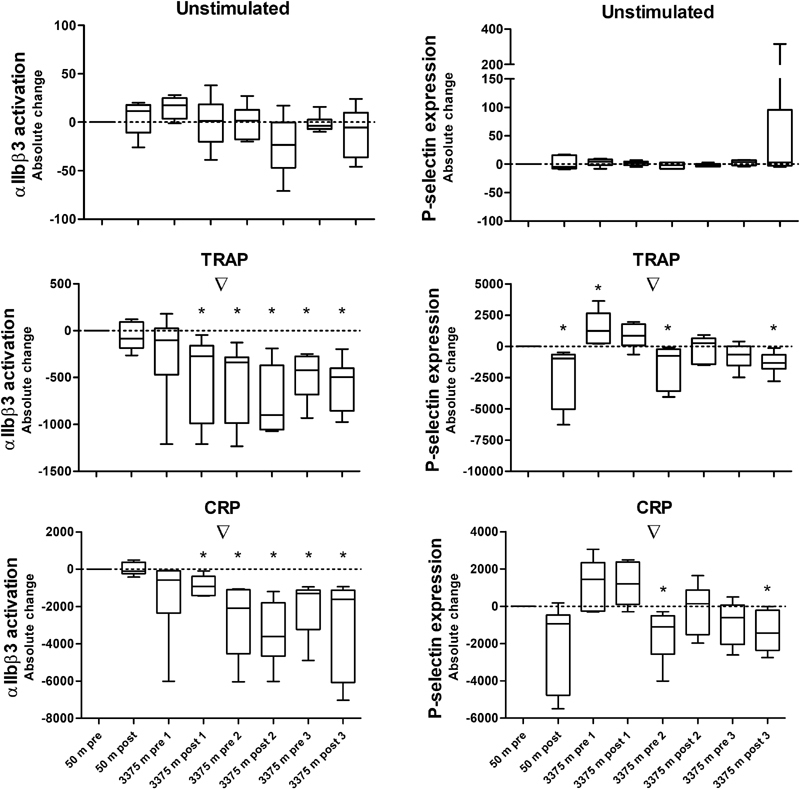
Influence of normoxic and hypoxic exercises on platelet activation. Platelet activation was measured before and after 2 hours of strenuous exercise, once at 50 m and three times at 3,375 m altitude. Platelets were stimulated with the agonist thrombin receptor-activating peptide (TRAP, 30 μmol/L, *middle panels* ) and collagen-related peptide (CRP, 5 μg/L, *bottom panels* ). An unstimulated condition was included as a control ( *top panels* ), showing no significant differences in baseline platelet activation between participants. Platelet activation was measured as αIIbβ3 activation and P-selectin expression in median fluorescence intensity. Results are expressed as the absolute change of 50 m pre-exercise; a value above 0 indicates an increase, and a value below 0 indicates a decrease. ▿ = *p* < 0.05 (Friedman's test); * = *p* < 0.05 compared with zero (Wilcoxon signed-rank test).

## Results

**Fig. 6:**
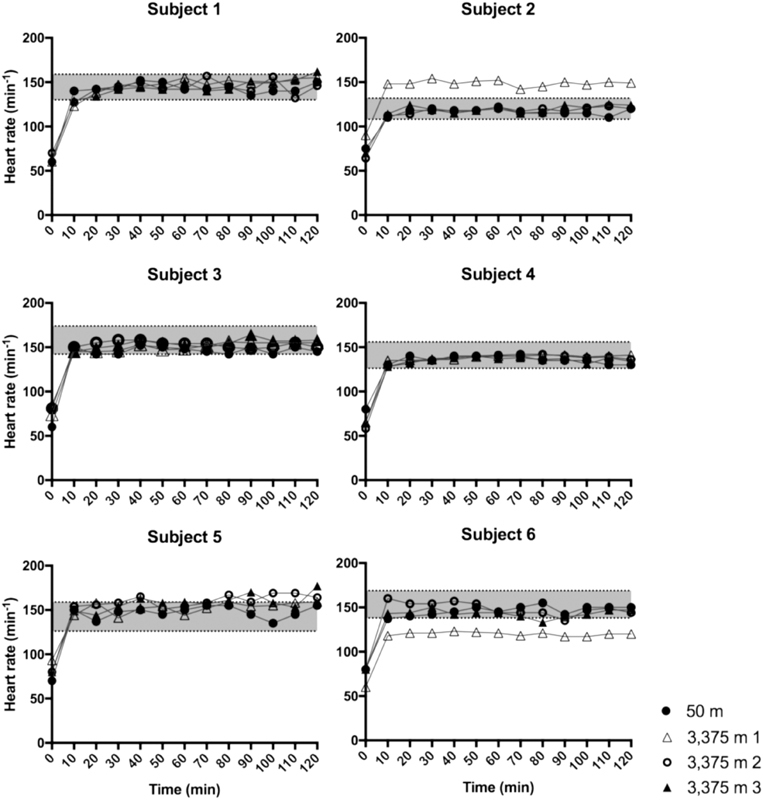
Individual heart rate traces during the exercise tests. Participants performed monitored exercise for 2 hours on four occasions: 1 time at 50 m altitude, and three times at 3,375 m altitude. Heart rate (HR, beats per minute) was noted every 10 minutes and participants were encouraged to keep their HR between 60 and 85% of their personal predefined HR reserve (HRR). The *gray area* on the graphs represents 60 to 85% of HRR.

Six trained adult men were recruited and passed the medical check-up (median age: 33.5 years, range: 18–48 years). All six participants completed the full study protocol. None of the participants experienced signs of AMS during the duration of the study (maximum Lake Louise AMS score: 3 points). As shown in Fig. 1 , the participants performed monitored exercise for 2 hours on four occasions: once at 50 m altitude, and three times at 3,375 m altitude. The participants cycled at an adequate exercise intensity for the majority of the time ( Fig. 6 ).

### Vital Signs, Biochemical Markers, Blood Count, and Coagulation Factor Levels

Results for vital signs are shown in Supplementary Table S3 , section A. The SpO _2_ dropped from 99% to 93% on day 1 at 3,375 m altitude, and to 92% on day 2 and 3, respectively. The resting HR increased slightly, from 56 to 64 per minute on day 1 at 3,375 m altitude.

Biochemical marker results are shown in Supplementary Table S3 , section B. Exposure to high altitude increased lactate levels slightly, yet nonsignificantly, and this effect was similar to exercise at sea level. Lactate levels did not rise any further due to the hypoxic exercise tests. Both normoxic and hypoxic exercises slightly increased the creatinine level, although it only rose significantly after normoxic exercise. Urea, albumin, and C-reactive protein levels did not change compared with baseline.

Blood count results are shown in Supplementary Table S3 , section C. Hb levels increased after acclimatization to high altitude, with a concurrent elevation in MCHC that was most prominent on day 2 at 3,375 m. Altitude itself did not have an effect on total WBC. RBC and Ht did not change due to the exercise tests or due to high altitude, except for day 2 pre-exercise. There was a consistent exercise-induced increase in total WBC, that depended on an increase in GR count, not LY or MO counts. This effect was present after both the normoxic and hypoxic exercise tests. The WBC returned to baseline levels every day. Altitude and/or exercise did not have a significant effect on PC, although there appeared to be a trend toward increased PC after each exercise test.

Coagulation factor level results are shown in Supplementary Table S3 , section D. Altitude itself caused a small, nonsignificant increase in baseline FVIII levels, without an increase in VWF or active VWF levels. The absolute change of VWF, active VWF, and FVIII compared with 50 m pre-exercise is shown in Fig. 3 . Repeated exercise caused a distinct zig-zag pattern in VWF, active VWF, and FVIII levels. VWF increased due to exercise, an effect that was most pronounced at sea level, and was less pronounced at day 2 and 3 at 3,375 m. VWF levels returned to baseline every day. Active VWF increased as well due to the exercise. The active VWF response was tapered after repeated hypoxic exercise. Both normoxic and hypoxic exercises increased the FVIII level, and FVIII did not fully return to baseline levels during the 3 days at high altitude. Fibrinogen and AT levels were neither affected by exercise, nor by high altitude.

### Effect of Normoxic Exercise on TG, Platelet Activation, and Clot Lysis Time

Results are expressed as the absolute change compared with 50 m pre-exercise; a value above 0 indicates an increase, and a value below 0 indicates a decrease. Effects of normoxic and hypoxic exercises on TG in WB and PRP are shown in Fig. 4 , and on TG in PPP are shown in Fig. 5 . The absolute values can be found in Supplementary Table S1 . There was no effect of normoxic exercise on WB TG peak (0 pM TF: *p* = 0.44; 0.5 pM TF: *p* = 0.81, compared with zero), nor on ETP (0 pM TF: *p >* 0.99; 0.5 pM TF: *p* = 0.63, compared with zero). In both PPP and PRP, normoxic exercise increased the peak (PPP CTI 1 pM TF: *p* = 0.03; 5 pM TF: *p* = 0.03; PRP: *p =* 0.03, compared with zero), but did not change the ETP (PPP 1 pM TF: *p >* 0.99; CTI 1 pM TF: *p* = 0.06; 5 pM TF: *p* = 0.44; PRP: *p* = 0.22, compared with zero).

Effects of normoxic and hypoxic exercises on platelet activation are shown in Fig. 6 . The absolute values can be found in Supplementary Table S2 . Baseline (unstimulated) platelet activation was not significantly different between participants under all test conditions (Friedman's αIIbβ3 activation: *p* = 0.062; P-selectin expression: *p* = 0.293). Normoxic exercise did not affect αIIbβ3 activation (CRP: *p >* 0.99; TRAP: *p* = 0.31, compared with zero), or CRP-induced P-selectin expression ( *p* = 0.06, compared with zero), but slightly decreased TRAP-induced P-selectin expression ( *p* = 0.03, compared with zero).

CLT was not affected significantly by the normoxic exercise ( Supplementary Table S3 , section E).

### Effect of Ascent to High Altitude on TG, Platelet Activation, and Clot Lysis Time

The ascent to 3,375 m altitude did not cause significant changes in the peak and ETP of WB TG ( Fig. 4 ). In PRP TG, the ETP was decreased at high altitude *(p =* 0.03, compared with zero *),* while the peak was decreased as well but did not reach significance *(p =* 0.06, compared with zero *)* . In PPP TG, at 5 pM TF the ETP was increased at high altitude ( *p* = 0.03, compared with zero), with no change in peak ( *p >* 0.99, compared with zero). PPP TG at 1 pM TF (peak and ETP both: *p* = 0.84, compared with zero) and CTI 1 pM TF (peak: *p* = 0.09 and ETP: *p* = 0.84, compared with zero) did not change due to the increased altitude ( Fig. 5 ).

Platelet αIIbβ3 activation did not change directly after ascent to high altitude (CRP: *p >* 0.99; TRAP: *p* = 0.22, compared with zero), whereas TRAP-induced P-selectin expression initially increased ( *p* = 0.03, compared with zero), as shown in Fig. 6 . Overall, the stay at high altitude depressed platelet αIIbβ3 activation triggered by both CRP and TRAP (CRP: Friedman's *p <* 0.0001; TRAP: Friedman's *p <* 0.001). The CRP-induced αIIbβ3 activation was maximally decreased on day 2 postexercise ( *p* = 0.03, compared with zero).

CLT was not affected significantly by altitude ( Supplementary Table S3 , section E).

### Effect of Repeated Hypoxic Exercise on TG, Platelet Activation, and Clot Lysis Time

Overall, in WB at 0 pM TF, TG peak (Friedman's *p* = 0.0028) as well as ETP (Friedman's *p* = 0.02) remained increased during the 3rd days at high altitude ( Fig. 4 ). There was no evident effect of repetitive hypoxic exercise on WB TG. Peak and ETP in PRP remained consistently decreased at high altitude (Friedman's *p* = 0.0003 and *p <* 0.0001, respectively). Hypoxic exercise appeared to further decrease PRP TG during the 3 test days at high altitude.

As shown in Fig. 5 , TG in PPP was not affected consistently by hypoxic exercise. Peak TG and ETP at 1 pM TF were overall not significantly different (Friedman's *p* = 0.19 and 0.30, respectively). However, there were some differences compared with 50 m pre-exercise. The peak at 5 pM TF was slightly increased after hypoxic exercise on day 2 and 3 (both *p* = 0.03 *,* compared with zero). Moreover, ETP at 5 pM TF remained slightly increased at high altitude (Friedman's *p* = 0.003), which was most prominent on day 3 (both pre- and postexercise: *p* = 0.03, compared with zero). Peak TG in CTI-anticoagulated PPP was slightly increased during day 2 and 3 at high altitude (Friedman's *p* = 0.01) and increased more postexercise on day 2 ( *p* = 0.03, compared with zero *)* .

There was no consistent exercise-dependent effect on platelet αIIbβ3 activation at high altitude ( Fig. 6 ). The depressed TRAP-induced P-selectin expression that was seen after normoxic exercise was not seen after hypoxic exercise on day 1 ( *p* = 0.16, compared with zero) and day 2 ( *p >* 0.99, compared with zero *)* , but was observed on day 3 ( *p* = 0.03, compared with zero). Likewise, postexercise P-selectin expression induced by CRP was unchanged at high altitude on the first 2 days ( *p* = 0.09 on day 1, *p >* 0.99 on day 2), but was significantly decreased on day 3 ( *p* = 0.03, compared with zero).

Overall, CLT did not change during the 3-day stay at high altitude ( Supplementary Table S3 , section E; Friedman's *p* = 0.6131).

## Discussion and Conclusion

In this pilot study, we examined the effects of normoxic and hypoxic strenuous exercises during 2 hours on TG, platelet activation, and fibrinolysis in six active and healthy men. The hypoxic exercise tests were performed on 3 consecutive days to determine whether hemostasis is modulated after repeated exercise in a hypoxic environment.

The participants were mildly hypoxic at 3,375 m altitude and acclimatized to the hypoxic environment at high altitude, by raising their Hb levels and MCHC after a 3-day stay at 2,473 m plus one night at 3,375 m altitude. Hb production increases in a hypoxic environment through elevation of erythropoietin, a response that generally takes around 4 days to become apparent. 32

Both normoxic and hypoxic exercises increased the GR count, which returned to baseline every day. Acute exercise is known to induce transient neutrophilia. 33 Neutrophils are the most abundant GRs and play an important role in destroying pathogenic invaders or cellular debris.

PC rose after the exercise tests, albeit nonsignificantly. Acute exercise is known to increase the number of platelets, which is thought to occur via mobilization of a splenic platelet pool through elevated catecholamines and/or shear stress. 12 34

We found no signs of dehydration after the exercise tests or due to the altitude, as Ht and albumin did not increase. 35

Normoxic exercise increased levels of VWF and active conformation VWF. The VWF level is known to be increased following exercise through release from endothelial Weibel–Palade bodies. 12 As a result of strenuous exercise, intravascular shear stress is increased and VWF is unfolded, exposing the A1 domain; this conformation is known as active VWF. 36 This active VWF binds to platelets more readily and is hence more thrombogenic. 37

Additionally, we found that normoxic exercise raised FVIII levels, an effect that is also well known. In the circulation, FVIII is bound to VWF, and therefore FVIII is most likely secondarily increased by VWF. 12 Moreover, normoxic exercise increased the peak TG, but not ETP in both PPP and PRP. Increased peak TG may be due to clotting activation mediated by contact factors, of which FVIII is one. 38

Recently, we performed another small-scale cycling study, in which five participants cycled 80 km at sea level on 3 consecutive days. 39 The normoxic cycling study also showed that exercise significantly increases VWF (antigen, propeptide, and active conformation) levels, FVIII levels, and peak TG. No increase in ETP was observed after cycling, which is in agreement with another large strenuous cycling study. 11 39

In the current study, the normoxic exercise slightly decreased TRAP-induced P-selectin expression and did not have an effect on αIIbβ3 activation in our active volunteers. In contrast with these findings, there is abundant data showing that physical exercise induces platelet activation. 12 However, there appears to be a different platelet response to exercise in physically trained subjects compared with sedentary subjects, as it has been found before that strenuous exercise does not induce platelet hyperreactivity in trained individuals. 40 Our subjects were trained, and it is possible that their platelets therefore did not become hyperreactive. Moreover, in the other small-scale cycling study, platelet P-selectin expression showed a similar decrease after exercise. 39

There was no effect of normoxic exercise on fibrinolysis in this study, as CLT did not change significantly. Short-term strenuous exercise is known to augment fibrinolysis through increased t-PA levels, which is released from endothelial cells, and decreased plasminogen activator inhibitor (PAI)-1 levels, the primary inhibitor of t-PA that is released from activated platelets. 12 The CLT is a global measurement for fibrinolysis, and may not be sensitive enough to pick up the exercise-induced hyperfibrinolysis.

We found that ascent to 3,375 m altitude mildly increased WB TG and FVIII levels, while it did not initially change platelet activation markers. However, on day 2 and 3 at high altitude, TG in PPP was slightly increased, and platelet αIIbβ3 activation and platelet-dependent TG were depressed. Hypoxia was previously found to decrease ex vivo platelet αIIbβ3 activation and aggregation. 19 In another recent high-altitude study performed by our group, we found that ascent to 3,883 m altitude increased TG but decreased platelet activation in healthy inactive volunteers. 13 However, in that study, the hypoxia was more profound and was accompanied by slightly increased lactate levels. Therefore, these two studies should not be compared one on one.

In this study, there was no effect of the high-altitude sojourn on fibrinolysis. Data on the effect of systemic hypoxia on fibrinolysis are scarce. In another study, it was found that hypobaric hypoxia equivalent to 2,438 m altitude does not change t-PA and PAI-1 levels. 41

We found that the hypoxic exercise tests elevated VWF, active VWF, and FVIII as well. Over the course of the 3 days at high altitude, the amplitude of exercise-induced elevation of VWF and active VWF decreased slightly, suggesting that either the VWF supply or the endothelial response to physical stress was diminishing.

In contrast to the exercise test at sea level, the hypoxic exercise tests did not have a consistent effect on peak TG. Platelet-dependent TG even appeared to show an exercise-related decrease. The diminished platelet αIIbβ3 activation at high altitude was not further aggravated by hypoxic exercise.

The findings from this hypoxic cycling study are in part similar to the findings of the other small-scale normoxic cycling study. In that study, the FVIII increase was tapered over 3 days, an effect that was also apparent by a decreasing amplitude of peak TG in plasma. Moreover, platelet P-selectin expression decreased after repeated exercise and did not recover fully, indicating exhaustion of the platelet response to repetitive exercise. 39 However, there is one major difference. In the current study, staying in a hypoxic environment appeared to further depress platelet αIIbβ3 activation and platelet-dependent TG.

We did not find an effect of repeated hypoxic exercise on fibrinolysis in the current pilot study. In the previous small-scale normoxic cycling study, CLT decreased very slightly on the first 2 days after exercise and decreased significantly on the third day. In another experimental study, it was found that severe hypoxia (equivalent to an altitude of 4,600 m) enhanced fibrinolytic activity after exercise, by decreasing the exercise-induced increase in PAI-1. 16

This study has some limitations. The small number of participants precluded the use of parametric statistics. Nonparametric statistical analyses such as the Friedman's test are quite conservative and are therefore relatively insensitive to clinically relevant differences. 42 For instance, the HR before and after exercise was not identified as statistically different, although they were clinically clearly different ( Fig. 2 and Supplementary Table S3 , section A). Therefore, we also used descriptive statistics to discover trends in the data, although this is more prone to biased interpretation.

While the percentage of maximum O _2_ consumption (%VO _2_ max) is considered the gold standard for standardization of exercise intensity, we used the percentage of HRR (%HRR). The relationship between %HRR and %VO _2_ max is not linear, especially at low exercise intensity and in subjects with low cardiorespiratory fitness, which limits its use in exercise physiology research. 23 However, the use of VO _2_ max in an environment with decreased atmospheric O _2_ pressure is of limited value. Acute hypoxia reduces the VO _2_ max, and despite increases in Hb and O _2_ saturation that can normalize arterial O _2_ content after acclimatization, VO _2_ max remains lower at high altitude. 16 32 Our goal was to standardize strenuous exercise both at sea level and high altitude. The %HRR is not affected by atmospheric O _2_ pressure, and is adequate for standardization of strenuous exercise. 23

We did not include any women in this study to minimize heterogeneity in our small group of participants. However, women are known to have a different hemostatic response to exercise, and therefore these results should not be extrapolated to them. 11 43 Moreover, there was a considerable age difference between the participants (18–48 years). Exercise tolerance and endothelial function are known to be affected by age, 44 hence this (in combination with the small sample size) has contributed to the relatively wide distributions of the data. All subjects were physically fit, exercising between 4 and 7.5 hours per week. Of note, four out of six subjects were regular cyclists. These variations could also potentially influence the response to our exercise protocol.

Taken together, the current data should be interpreted with caution. A large follow-up study is necessary for identifying differences that are both clinically and statistically relevant, and to investigate if the effect is present in women as well.

## Conclusion

Strenuous exercise induces a procoagulant phenotype that is mediated by the endothelium, by increasing VWF and secondarily raising FVIII levels. The amplitude of the endothelial response to exercise decreases after repetitive exercise. A hypoxic environment may further increase the exhaustive effect of repetitive exercise on hemostasis by depressing platelet aggregation potential and platelet-dependent TG. These results warrant further investigations, to determine how much time is required for the endothelial response to exercise to recover and whether platelet activation is depressed more during prolonged exposure to a hypoxic environment.
