# Supplementary material for: A Hypoxic Environment Attenuates Exercise-Induced Procoagulant Changes Due to Decreased Platelet Activation
Source: TH Open. 2019 Jul 22;3(3):e216–26. doi: 10.1055/s-0039-1692991 (PMC6645913; doi:10.1055/s-0039-1692991)
Supplement: Supplementary file 1 — Supplementary Material [file 10-1055-s-0039-1692991-s190024.pdf]

Supplementary Table S1 Thrombin generation

	50 m altitude		3,375 m altitude						*p*-Value
	Pre	Post	Pre 1	Post 1	Pre 2	Post 2	Pre 3	Post 3	
A. WB 0 pM TF									
Peak (nM)	0	+4.5 [−3.2; +21]	+27 [+9.0; +42]	+41 [+30; +50]	+31 [+24; 39]	+54 [+21; +59]	+16 [+10; +43]	+28 [+14; +52]	0.0028
ETP (nM·min)	0	+35 [−40; +38]	+157 [+42; +177]	+130 [+116; +167]	+39 [+7.1; +93]	+122 [+7.4; +173]	+5.9 [−11; +166]	+3.3 [−9.6; +73]	0.0202
B. WB 0.5 pM TF									
Peak (nM)	0	−4.0 [−13; +9.8]	+3.4 [−17; +7.4]	−8.2 [−24; +17]	+5.5 [−9.7; +15]	−1.33 [−8.9; +32]	+14 [−5.3; +39]	+0.8 [−2.6; +9.8]	0.6762
ETP (nM·min)	0	−11 [−67; +39]	+112 [−1.8; +151]	+81 [+45; +170]	+53 [−15; +84]	+84 [+20; +181]	+55 [−2.9; +195]	+11 [−6.6; +39]	0.0387
C. PRP									
Peak (%)	0	**+23**[a] [+15; +49]	−43 [−59; −29]	**−58**[a] [−96; −13]	−13.85 [−51; −2.8]	−26 [−41; +14]	−30 [−42; +9.1]	−39 [−73; −14]	0.0003
ETP (%)	0	+4.1 [−0,8; +19]	**−41**[a] [−62; −28]	**−58**[a] [−73; −26]	−28 [−54; −15]	**−41**[a] [−52; −20]	**−30**[a] [−41; −7.5]	**−50**[a] [−62; −21]	<0.0001
D. PPP 1 pM TF									
Peak (%)	0	+20 [+3.7; +50]	−2.3 [−13; +42]	+26 [−7.8; +63]	−6.8 [−26; +3.3]	+1.4 [−18; +22]	−0.3 [−13; +10]	+15 [−3.6; +47]	0.1854
ETP (%)	0	+8.4 [−47; +17]	−4.0 [−14; +8.7]	+4.4 [−17; +26]	−6.5 [−29; +0.9]	−3.9 [−28; +9.8]	+6.3 [−0.1; +11]	+12 [−2.5; 23]	0.2950
E. CTI-PPP 1 pM TF									
Peak (%)	0	**+17**[a] [+9.2; +55]	−8.2 [−17; −0.8]	+6.9 [−4.8; +70]	+6.4 [−11; +27]	**+15**[a] [+6.3; +29]	+14 [+0.7; +23]	+18 [+6.0; 71]	0.0125
ETP (%)	0	+12 [+3.9; +65]	+1.1 [−10.03; +25]	+4.1 [−2.6; +65]	+6.8 [−8.2; +44]	+10 [+3.1; +43]	**+18**[a] [+7.0; +44]	**+11**[a] [+0.9; +62]	0.0970
F. PPP 5 pM TF									
Peak (%)	0	**+18**[a] [+13; +36]	−0.1 [−7.0; +11]	+9.1 [+0.1; +22]	−1.1 [−10; +8.2]	**+18**[a] [+3.1; +39]	+10 [−2.7; +24]	**+17.2**[a] [+6.0; +32]	0.0013
ETP (%)	0	−2.0 [−5.9; +3.1]	**+6.8**[a] [+4.9; +12]	−2.0 [−6.0; +3.1]	+11 [+2.4; +14]	+7.6 [+1.6; +14]	**+11**[a] [+7.5; +13]	**+11**[a] [+3,0; +14]	0.0027

A. Thrombin generation (TG) in whole blood (WB) without added tissue factor (TF). Parameters derived from the TG curve are peak height (peak in nM) and endogenous thrombin potential (ETP in nM·min).
B. TG in WB triggered with 0.5 pM TF. Parameters derived from the TG curve are peak in % of normal pool plasma (NPP) and ETP in % of NPP.
C. TG in platelet-rich plasma (PRP) triggered with 1 pM TF. Parameters derived from the TG curve are peak in % of NPP and ETP in % of NPP.
D. TG in platelet poor plasma (PPP) triggered with 1 pM TF. Parameters derived from the TG curve are peak in % of NPP and ETP in % of NPP.
E. TG in PPP anticoagulated with corn trypsin inhibitor (CTI) triggered with 1 pM TF. Parameters derived from the TG curve are peak in % of NPP and ETP in % of NPP.
F. TG in PPP triggered with 5 pM TF. Parameters derived from the TG curve are peak in % of NPP and ETP in % of NPP.
Note: TG was measured before and after 2 hours of strenuous exercise, once at 50 m altitude and three times at 3,375 m altitude. $N = 6$, one measurement is missing (subject 5, WB TG post 3). Results are expressed as the absolute change of 50 m pre-exercise; a value above 0 indicates an increase, a value below 0 indicates a decrease. Data are presented as median [25th percentile; 75th percentile]. *p*-Value = Friedman's test.
[a] $p < 0.05$ compared with zero (Wilcoxon signed-rank test).

Supplementary Table S2 Platelet activation

	50 m altitude		3,375 m altitude						*p*-Value
	Pre	Post	Pre 1	Post 1	Pre 2	Post 2	Pre 3	Post 3	
A. αIIbβ3 activation									
Unst.<br>(MFI)	0	+11.5<br>[−11.0; + 17.8]	+17.5<br>[+3.5; + 25.0]	+1.0<br>[−20.3; + 18.5]	+1.5<br>[−17.8; + 12.8]	−23.5<br>[−47.0; −0.3]	−4.0<br>[−7.0; + 2.5]	−5.5<br>[−36.3; + 9.8]	0.062
CRP<br>(MFI)	0	−114.5<br>[−239.0; + 373.8]	−587.5<br>[−2,365; −75.0]	−**919.5**[a]<br>[−1,414; −385.8]	−**2,097**[a]<br>[−4,542; −1,101]	−**3,612**[a]<br>−4.675; −1,798]	−**1,305**[a]<br>[−3,232; −1,118]	−**1,610**[a]<br>[−6,070; −1.139]	<0.001
TRAP<br>(MFI)	0	−85.0<br>[−184.3; + 93.5]	−101.5<br>[−470.8; + 27.0]	−**273.0**[a]<br>[−991.3; −159.5]	−**338.5**[a]<br>[−985.8; −282.8]	−**897.5**[a]<br>[−1,057; −370.0]	−**422.5**[a]<br>[−681.5; −274.0]	−**497.5**[a]<br>[−854.3; −401.0]	<0.001
B. P-selectin expression									
Unst.<br>(MFI)	0	−4.5<br>[−7.5; +16.25]	+4.5<br>[−2.0; +8.5]	+2.0<br>[−2.0; + 4.8]	−1.5<br>[−8.0; +3.0]	−1.5<br>[−4.0; +0.8]	+4<br>[−2.5; +7.3]	+3<br>[−2.8; 95.5]	0.293
CRP<br>(MFI)	0	−941.5<br>[−4,774; −466.8]	+1,434<br>[−259.9; + 2,350]	+1,193<br>[+106.3; + 2,375]	−**1,108**[a]<br>[−2.572; −515.0]	+126.0<br>[−1,515; + 864.8]	−615.0<br>[−2048; + 54.5]	−**1,436**[a]<br>[−2,361; −204.5]	<0.001
TRAP (MFI)	0	−**977.0**[a]<br>[−5,042; −660.8]	+**1,240**[a]<br>[+225.0; + 2,633]	+829.0<br>[+62.5; + 1,787]	−**760.0**[a]<br>−3,582; −206.5]	+252.5<br>[−1,448; + 648.5]	−665.0<br>[−1,545; + 22.8]	−**1,351**[a]<br>[−1,795; −678.3]	<0.001

Abbreviation: Unst., unstimulated platelets.

Note: Platelet activation was measured as αIIbβ3 activation and P-selectin expression. Blood was drawn before and after 2 hours of strenuous exercise, once at 50 m altitude and three times at 3,375 m altitude. As agonists, collagen-related peptide (CRP, final concentration 5 μg/L) and thrombin receptor activating peptide (TRAP, final concentration 30 μmol/L) were used. An unstimulated condition was included as control. Results are expressed as the absolute change of 50 m pre-exercise; a value above 0 indicates an increase, a value below 0 indicates a decrease. Data are presented as median [25th percentile; 75th percentile], $p < 0.05$ was considered statistically significant. *p*-value = Friedman's test.

[a] $p < 0.05$ compared with zero (Wilcoxon signed-rank test).

Supplementary Table S3 Vital signs, biochemical markers, blood count, coagulation factor levels, and clot lysis time

	50 m altitude		3,375 m altitude						*p*-Value
	Pre	Post	Pre 1	Post 1	Pre 2	Post 2	Pre 3	Post 3	
**A. Vital signs**									
SpO_2	99 [1]	98 [2]	93 [3]	**90**[a] [4]	92 [3]	**91**[a] [7]	**92**[a] [2]	92 [3]	0.0004
HR	56 [13]	148 [24]	64 [17]	**152**[a] [20]	59 [13]	145 [21]	69 [13]	**153**[a] [34]	<0.0001
**B. Biochemical markers**									
Lactate	1.63 [0.33]	2.17 [1.04]	2.16 [0.84]	2.1 [0.24]	1.41 [0.24]	1.98 [0.62]	1.67 [0.66]	1.91 [0.62]	0.0035
Creatinine	68.7 [13.3]	**74.6**[a] [13.8]	69.8 [19.6]	72.5 [14.5]	68.0 [16.1]	70.9 [11.1]	69 [13.4]	73.4 [8.23]	0.0074
Urea	5.3 [1.38]	5.9 [1.35]	4.95 [1.15]	4.65 [0.75]	4.75 [1.33]	4.95 [1.27]	5.0 [0.78]	4.65 [1.1]	0.0124
Albumin	35.4 [2.8]	38.1 [5.3]	36.4 [4.7]	38.1 [4.3]	35.9 [3.0]	36.4 [4.7]	33.45 [1.1]	35.85 [4.4]	0.0073
CRP	0.45 [0.78]	0.55 [0.82]	0.45 [2.0]	0.4 [1.88]	0.7 [1.1]	0.6 [1.25]	0.7 [1.43]	0.75 [1.4]	0.0155
**C. Blood count**									
RBC	4.7 [0.7]	4.8 [0.8]	5.2 [0.3]	5.1 [0.6]	**5.4**[a] [0.3]	5.3 [0.2]	4.9 [0.5]	5.1 [0.6]	0.0026
Ht	0.42 [0.04]	0.42 [0.05]	0.45 [0.02]	0.44 [0.02]	**0.46**[a] [0.03]	0.45 [0.03]	0.44 [0.04]	0.44 [0.02]	0.0117
Hb	8.2 [0.4]	8.5 [1.4]	10.0 [0.6]	**10.1**[a] [1.0]	**10.2**[a] [0.8]	**10.0**[a] [1.0]	9.6 [0.8]	9.8 [0.7]	<0.0001
MCHC	19.9 [0.9]	20.1 [0.6]	21.9 [1.1]	**22.8**[a] [1.4]	**22.5**[a] [0.9]	22.4 [1.0]	22.1 [0.6]	22.4 1.3]	<0.0001
WBC	5.6 [2.4]	7.2 [1.3]	6.5 [2.0]	**8.0**[a] [3.6]	7.3 [1.8]	**8.6**[a] [4.0]	6.7 [1.7]	7.9 [2.5]	0.0016
Gr	3.4 [2.1]	4.8 [1.3]	4.0 [1.3]	**5.3**[a] [2.7]	4.8 [2.0]	**5.4**[a] [2.7]	4.4 [1.5]	**5.4**[a] [2.1]	0.0002
LY	2.2 [0.9]	2.1 [1.2]	2.4 [0.9]	2.1 [0.9]	2.1 [0.4]	2.1 [1.5]	2.1 [0.6]	2.3 [1.1]	0.7734
MO	0.3 [0.1]	0.3 [0.2]	0.4 [0.2]	0.4 [0.1]	0.4 [0.2]	0.4 [0.2]	0.3 [0.3]	0.3 [0.2]	0.6597
PC	238 [60]	267 [85]	251 [60]	268 [84]	229 [57]	281 [63]	234 [41]	264 [59]	0.0629
**D. Coagulation factor levels**									
VWF:Ag	93 [61]	**152**[a] [86]	89 [68]	142 [78]	99 [88]	125 [83]	93 [76]	121 [87]	0.0002
aVWF	124 [32]	**173**[a] [117]	131 [43]	**197**[a] [61]	148 [68]	**177**[a] [87]	127 [54]	148 [53]	0.0006
FVIII:C	108 [18]	**161**[a] [97]	114 [33]	163 [112]	116 [39]	164 [40]	116 [41]	154 [48]	0.0009
Fg	2.6 [1.1]	2.6 [1.6]	2.8 [1.2]	2.7 [1.1]	2.9 [1.2]	2.7 [1.3]	2.7 [1.1]	2.8 [1.2]	0.0923
AT	95 [15]	99 [13]	98 [10]	97 [14]	96 [9]	101 [16]	93 [9]	95 [6]	0.1536
**E. Fibrinolysis**									
CLT	24.0 [16.8]	26.0 [18.4]	26.7 [19.2]	26.3 [15.2]	25.8 [20.0]	26.0 [19.0]	23.5 [15.4]	23.5 [16.6]	0.6131

A. Vital signs: peripheral oxygen saturation (SpO_2, %), heart rate (HR, beats/min).
B. Biochemical markers: lactate (mmol/L), creatinine (μmol/L), urea (mmol/L), albumin (g/L) and C-reactive protein (CRP, mg/L).
C. Blood count: red blood cell count (RBC, $\times 10^{12}$/L), hematocrit (Ht, L/L), (hemoglobin (Hb, mmol/L), mean corpuscular hemoglobin concentration (MCHC, mmol/L), white blood cell count (WBC, $\times 10^{9}$/L), granulocyte count (Gr, $\times 10^{9}$/L), lymphocyte count (LY, $\times 10^{9}$/L), monocyte count (MO, $\times 10^{9}$/L), platelet count (PC, $\times 10^{9}$/L).
D. Coagulation factor levels: von Willebrand Factor antigen (VWF:Ag, %), VWF in active conformation (aVWF, % of normal pool plasma), factor VIII concentration (FVIII:C, %), fibrinogen levels (Fg, g/L), antithrombin (AT, %).
E. Fibrinolysis: clot lysis time (CLT, min), defined as the time from half-maximum clot formation to half-maximum clot degradation.
Note: All parameters were measured at 50 m and 3,375 m altitude, before and after exercise ($n = 6$), and are expressed as median [interquartile range]. *p*-Value = Friedman's test, $p < 0.05$ was considered statistically significant.
[a] $p < 0.05$ compared with 50 m pre-exercise (Dunn's post-hoc analysis).
